# Machine learning identifies novel signatures of antifungal drug resistance in *Saccharomycotina* yeasts

**DOI:** 10.1371/journal.pgen.1012091

**Published:** 2026-03-17

**Authors:** Marie-Claire Harrison, David C. Rinker, Abigail L. LaBella, Dana A. Opulente, John F. Wolters, Xiaofan Zhou, Xing-Xing Shen, Marizeth Groenewald, Chris Todd Hittinger, Antonis Rokas

**Affiliations:** 1 Department of Biological Sciences and Evolutionary Studies Initiative, Vanderbilt University, Nashville, Tennessee, United States of America; 2 Department of Bioinformatics and Genomics, University of North Carolina at Charlotte, Kannapolis, North Carolina, United States of America; 3 Center for Computational Intelligence to Predict Health and Environmental Risks (CIPHER), University of North Carolina at Charlotte, Charlotte, North Carolina, United States of America; 4 Laboratory of Genetics, DOE Great Lakes Bioenergy Research Center, Center for Genomic Science Innovation, J. F. Crow Institute for the Study of Evolution, Wisconsin Energy Institute, University of Wisconsin-Madison, Madison, Wisconsin, United States of America; 5 Department of Biology, Villanova University, Villanova, Pennsylvania, United States of America; 6 Guangdong Province Key Laboratory of Microbial Signals and Disease Control, Integrative Microbiology Research Center, South China Agricultural University, Guangzhou, China; 7 Zhejiang Key Laboratory of Biology and Ecological Regulation of Crop Pathogens and Insects, Institute of Insect Sciences, Zhejiang University, Hangzhou, China; 8 Westerdijk Fungal Biodiversity Institute, Utrecht, The Netherlands; University of Georgia, UNITED STATES OF AMERICA

## Abstract

Antifungal drug resistance is a major challenge in fungal infection management. Numerous genomic changes are known to contribute to acquired drug resistance in clinical isolates of specific pathogens, but whether they broadly explain natural resistance across entire lineages is unknown. We leveraged genomic, ecological, and phenotypic trait data from naturally sampled strains from nearly all known species in subphylum *Saccharomycotina* to examine the evolution of resistance to eight antifungal drugs. The phylogenetic distribution of drug resistance varied by drug; fluconazole resistance was widespread, while 5-fluorocytosine resistance was rare, except in *Lipomycetales*. A random forest algorithm trained on genomic data predicted drug-resistant yeasts with 54–75% accuracy. Fluconazole resistance was consistently predicted with the highest accuracy (75.2%). Furthermore, fluconazole resistance prediction accuracy was similar between models trained on genome-wide variation in the presence and number of InterPro protein annotations across *Saccharomycotina* (75.2%) and those trained on amino acid sequence alignment data of Erg11, a protein known to be involved in fluconazole resistance (74.3-74.9%). Interestingly, the top Erg11 residues for predicting fluconazole resistance across *Saccharomycotina* do not overlap with, are not spatially close to, and are less conserved than those previously linked to resistance in clinical isolates of *Candida albicans*. *In silico* deep mutational scanning of the *C. albicans* Erg11 protein reveals that amino acid variants implicated in clinical cases of resistance are almost universally destabilizing while variants in our most informative residues are energetically more neutral, explaining why the latter are much more common than the former in natural populations. Importantly, previous experimental analyses of *C. albicans* Erg11 have shown that amino acid variation in our most informative residues, despite having never been directly implicated in clinical cases, can directly contribute to resistance. Our results suggest that studies of natural resistance in yeast species never encountered in the clinic will yield a fuller understanding of antifungal drug resistance.

## Author summary

Resistance to drugs is a major challenge in the treatment of fungal infections. Many fungi are naturally resistant to antifungal drugs, but the genetic variants involved are poorly characterized. We employed machine learning, structural, and evolutionary approaches to identify genetic variants associated with drug resistance across an ancient yeast lineage. By focusing on a protein known to be involved in resistance to the antifungal drug fluconazole, we identified several novel variants that were significantly associated with drug resistance, but whose evolutionary and biophysical properties differ from previously characterized clinical variants in specific human pathogens. Furthermore, previous *in vitro* experimental analyses have shown that several of these natural variants can directly contribute to resistance. We suggest that studies on the genetic basis of drug resistance across entire fungal lineages can complement studies of human pathogenic fungi, leading to fuller understanding of the drug resistance challenge.

## Introduction

Yeasts in the subphylum *Saccharomycotina* (hereafter referred to as yeasts) are genomically diverse, geographically widely distributed, and found in diverse habitats [1]. Opportunistic pathogens in this subphylum are a significant global health concern (WHO, 2022), especially for patients with compromised immune systems, for various reasons including for their resistance to antifungal drugs [2–4]. For example, initially susceptible strains of *Candida albicans* and *Nakaseomyces glabratus* syn. *Candida glabrata* can quickly evolve (or acquire) resistance to antifungal drugs in clinical settings [5,6]. Moreover, strains of other pathogens, most notably strains of the emerging pathogen *Candidozyma auris* (formerly *Candida auris*) can also rapidly acquire resistance [7,8]. Susceptibility screens for antifungal drugs in hundreds of *Saccharomycotina* species have further revealed that a substantial percentage of species are naturally resistant [9]. However, even though the genetic variants that underlie evolved resistance in clinical settings have been extensively characterized [10–14], natural genetic variants implicated in antifungal drug resistance are poorly understood.

There are three major classes of antifungal drugs, namely echinocandins (e.g., caspofungin and micafungin), azoles (e.g., fluconazole, voriconazole, and itraconazole), and polyenes (e.g., amphotericin B), and two minor classes, allylamines (e.g., terbinafine) and nucleoside analogs (e.g., 5-fluorocytosine) [2,15–18]. Resistance to each class has been observed in clinical settings, including observations of pathogens that are resistant to multiple different drugs [2,10,17,19,20]. Elucidating the genetic variants that confer antifungal drug resistance is a crucial step in the development of effective treatment of these pathogens.

One of the main targets of azoles, polyenes, and allylamines is the ergosterol synthesis pathway, with resistance in clinical isolates typically conferred through mutations in genes of the pathway. For example, fluconazole resistance in clinical isolates of *C. albicans* is often mediated through mutations in the *ERG11* gene, which encodes a lanosterol 14-alpha-demethylase [11,12]. Fluconazole strongly binds the active site of Erg11, inhibiting its ability to biosynthesize ergosterol, the primary sterol of the fungal cell membrane [21,22]. Resistance to azoles can also arise through regulatory changes that may either increase the expression of Erg11 (e.g., via Upc2 [23,24], or that upregulate cellular efflux pathways (e.g., ATP-binding cassette family and the major facilitator superfamily [17,20]) to actively remove the drugs. Polyenes also target the ergosterol biosynthesis pathway by binding directly to ergosterol, disrupting membrane stability [2,15]. Similarly, terbinafine is an allylamine antifungal that inhibits Erg1 (squalene epoxidase) activity, which also causes a lack of ergosterol and disrupts membrane stability in yeasts [25]. Resistance to these drugs most often stems from mutations in the drug target(s) that reduce or prevent drug binding (e.g., Erg1) or lead to the production of alternate sterols [2,25,26]. However, these resistance mechanisms often come with severe tradeoffs for membrane stability in yeasts, so resistance to these antifungals is not common [2,25].

Echinocandins target the cell wall, inhibiting synthesis of β-glucans by binding to the Fks1 and Fks2 proteins, which are important for yeast cell wall resilience [2]. However, mutations in *FKS1* and *FKS2* that prevent echinocandin binding can reduce drug efficacy [2]. Finally, 5-fluorocytosine is a nucleoside analog that targets fungal pathogens by inhibiting RNA and DNA synthesis in fungi [15,18]. Resistance occurs either by decreased uptake of the drug, or loss of one of the pyrimidine salvage enzymes, which convert 5-fluorocytosine to 5-fluorouridylic acid, the active state of the drug inside the fungal cell [15,18].

Most studies of antifungal drug resistance have been examinations of drug-resistant clinical isolates [12–14,23–25,27–30]. Such studies have been foundational to our understanding of the evolution of antifungal drug resistance and of the genes and pathways involved, but several questions remain. How widespread is natural resistance across entire clades? And are the genes and mutations that are associated with natural resistance the same as those that confer resistance in the clinic?

To answer these two questions, we integrated data from the Y1000 + Project (http://y1000plus.org) encompassing genomic, ecological, and metabolic profiles of over 1,000 yeast species [1,31], with experimental measurements of drug resistance against eight clinically relevant, antifungal drugs for 532 yeast species [9] and previous experimental deep mutational scanning data of *C. albicans* Erg11 to azole antifungal drugs [27] and *in silico* deep mutational scanning data of Erg11 proteins across *Saccharomycotina*. Analysis of our data using machine learning, structural biology, and phylogenetic approaches provided us with a novel window to understanding both the biology of drug resistance (in non-clinical settings) as well as how machine learning models complement structural and evolutionary approaches for identifying drug resistance-associated genetic variants.

## Results

### The distribution of drug resistance varies across the *Saccharomycotina* yeast phylogeny

**Fig 1:**
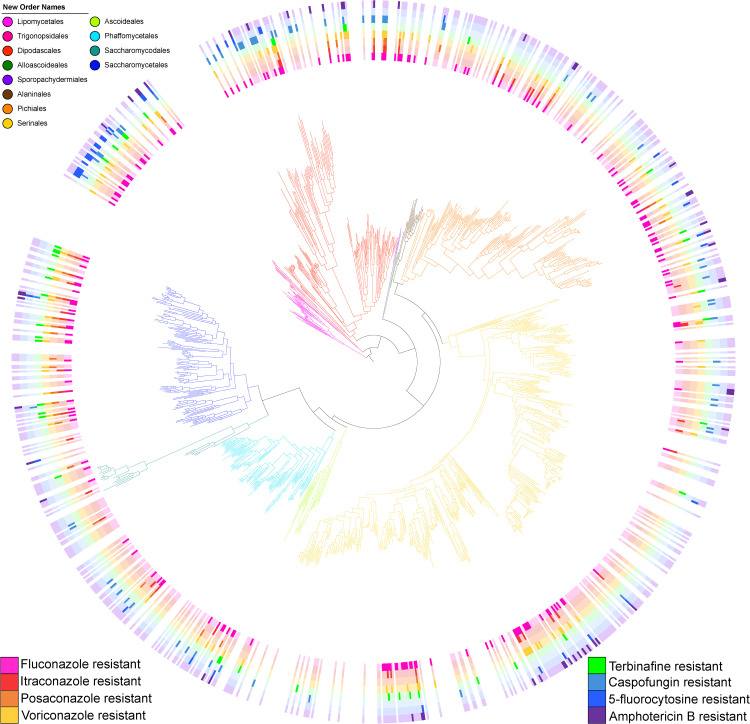
Resistance profiles to antifungal drugs vary throughout the *Saccharomycotina* subphylum. **Resistance to some drugs is lineage-specific (e.g., 5-fluorocytosine resistance), but resistance to others is broadly distributed (e.g., fluconazole resistance).** Dark colors denote resistance, light colors denote susceptibility, and no color denotes absence of testing. Yeast species names are omitted for easier visualization, but they can be found in S4 Fig. The colors of the different branches of the phylogeny correspond to the 12 taxonomic orders [1,83]. Drug resistance data obtained using the microdilution technique described in Desnos-Ollivier et al. [9]. Note that this visualization of antifungal drug resistance profiles does not consider within-species variation in drug resistance. There are 1,154 strains in the phylogeny, 532 with known values of resistance to antifungals.

To examine patterns of evolution of resistance, we plotted the resistance of 532 yeast species to eight different antifungal drugs [9] on the yeast phylogeny (Fig 1) [1]; the majority of strains were natural or environmental isolates (494 of 532 or 93%) with only 38 out of the 532 (7%) isolated from mammalian-associated environments (S1 Table). The antifungal resistance profiles of these mammalian-associated yeasts did not significantly differ from the rest of the dataset for any antifungal drug (S1 Table). Resistance to fluconazole was by far the most common, with 34.2% (182/532) of species tested being resistant (S2 Table). Resistance to voriconazole was the next most frequent (92/532 or 17.2%), followed by caspofungin (69/532 or 13.0%), amphotericin B (53/532 or 9.8%), itraconazole (46/532 or 8.6%), terbinafine (42/532 or 7.9%), 5-fluorocytosine (41/532 or 7.7%), and posaconazole (33/532 or 6.2%) (S2 Table). Out of 264 yeasts with resistance to any drug, over half (148) were resistant to two or more drugs. Of the yeasts that were resistant to a single drug, 42.2% (49/116) were resistant to fluconazole (S2 Table). We note that each species in our dataset is represented by a single strain. Since differences in resistance between strains of pathogenic yeasts have been observed [32–34], the resistance phenotype recorded for the strains examined in our study may not be always representative of the entire species.

In general, resistance to any of the eight antifungal drugs was rare in the 111 yeasts in the order *Serinales* (which includes the genus *Metschnikowia*, *C. auris*, as well as *C. albicans* and its relatives) (S3 Table). Resistance to 5-fluorocytosine was rare outside of the *Lipomycetales* and *Trigonopsidales* orders, while caspofungin resistance was rare only within the *Serinales*, *Pichiales*, and *Saccharomycetales*. In contrast, resistance to the azoles (including fluconazole), terbinafine, and amphotericin B tended to be relatively evenly distributed throughout the phylogeny, with only a few exceptions (S3 Table).

Notably, using the D measure developed by Fritz and Purvis [35], we found that the distributions of resistance to different antifungal drugs across the yeast phylogeny were neither randomly distributed nor were explained entirely by evolutionary history (Table 1). This pattern of sporadic resistance suggests that resistance to these drugs (or functional analogs of these drugs) repeatedly arose during yeast evolution and was likely adaptive. Both the broad distribution and the repeated evolution of antifungal resistance are particularly surprising, considering that 93% of yeast species examined are represented by natural isolates and have never been observed in the clinic (S1 Table).

**Table 1:** The distributions of resistance to antifungal drugs are not explained by evolutionary history.

Drug	Counts of resistance states (not resistant: resistant)	Estimated D (Fritz and Purvis)	Probability of D estimate resulting from:	
			No (random) phylo.structure	Brownian phylo. structure
Fluconazole	348:182	0.487778	0.0000	0.0000
Caspofungin	461:69	0.5021695	0.0000	0.0000
Any azole	335:195	0.5768153	0.0000	0.0000
Amphotericin B	478:52	0.5805753	0.0000	0.0000
Voriconazole resistance	438:92	0.6960791	0.0000	0.0000
Terbinafine resistance	488:42	0.7827477	0.0030	0.0000
Itraconazole resistance	484:46	0.8587058	0.0290	0.0000

### A random forest algorithm identifies gene and sequence features predictive of resistance

To identify genomic, phenotypic, and ecological features linked to the repeated evolution of drug resistance, we trained a random forest algorithm on genomic, metabolic growth, and isolation environment data from the Y1000 + Project [1,31]. Training on metabolic growth and isolation environment data yielded accuracies of 54–75% (average 63%) and 47–63% (average 55%), respectively (S4 Table). The features that, on average, most contributed to accuracy for resistance across all the drugs tested were growth on salicin and cellobiose for the models trained on metabolic data, while Arthropoda animal type and having a microbe association were the most informative traits for models trained on isolation environment data (S5 Table). The highest accuracy values were obtained when predicting resistance to 5-fluorocytosine in models trained on metabolic data, largely because there are numerous growth substrates (likely unrelated to drug resistance) that show the same clade-specific distribution as 5-fluorocytosine (S4 Table). The metabolic and environmental features that most contributed to accuracy for fluconazole resistance were growth on glucosamine and isolation from grasses, respectively (S1 Fig).

**Fig 2:**
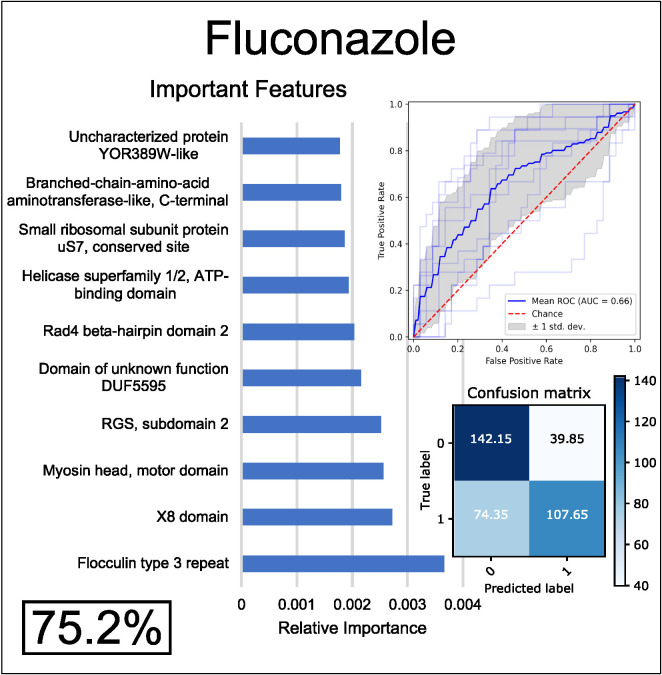
A random forest algorithm predicts resistance to fluconazole with moderate accuracy from variation in InterPro functional annotations (n = 532). Accuracy is shown in the form of cross-validated balanced accuracy over 20 down-sampled runs (value insight rectangle in bottom left of each panel). The confusion matrix (bottom right) shows yeasts predicted correctly to be sensitive (true negatives, top left), yeasts predicted to be resistant but are not (false positives, top right), yeasts correctly predicted to be resistant (true positives, bottom right), and yeasts correctly predicted to be sensitive (false negatives, bottom left). The Receiver Operating Characteristic (ROC) curve (upper right) shows the true positive rate over false positive rate with changing classification thresholds. The feature importance graph (left) shows the InterPro annotations that are most informative for predicting resistance to each drug. Note that the most informative genomic features were not linked to known drug resistance genes.

When trained on genomic data (i.e., on variation in InterPro functional annotations across the genomes of species), models predicted resistance to each of the eight antifungals with ~54–75% accuracy (Figs 2, S2). Fluconazole resistance was predicted most accurately (75.2%) and itraconazole resistance was predicted least accurately (53.1%) (Figs 2, S2). Although all the azoles belong to the same drug class, their level of correlation across yeasts was not very high as the numbers of yeasts resistant to posaconazole and itraconazole were considerably lower than those to fluconazole—only 19 yeasts were resistant to all 4 azoles while 195 yeasts were resistant to one or more azoles. However, resistance to voriconazole was more common and had higher overlap with fluconazole resistance—87/92 species that were resistant to voriconazole were also resistant to fluconazole. Anticipating that the higher accuracy in predicting fluconazole resistance would afford the best potential to uncover insights into the mechanisms of the evolution of drug resistance, we chose to focus on fluconazole resistance going forward.

The most well-characterized genomic determinants of azole resistance in the major human pathogen *C. albicans* are non-synonymous variants in Erg11. Erg11 is the drug target of the azole class of drugs, and resistance can arise by both mutations that impede the drug’s ability to bind to the protein, as well as copy number variants of *ERG11* or its regulators [10–13,29,36–41]. Therefore, we expected to see this gene (or other genes in the ergosterol pathway) in the top features of this model. However, we found that the top features for predicting fluconazole resistance implicated neither Erg11, which ranked 129^th^ in prediction importance, nor other genes in the ergosterol pathway.

Rather, the most informative genomic features were linked to cell wall-associated functional annotations. The flocculin type 3 repeat (IPR025928; the top feature), which is found in diverse proteins, including in the flocculation proteins Flo5, Flo9, and Flo10 in *S. cerevisiae* [42] (Fig 2), mediates cell-cell adhesion and the formation of multicellular clumps, also called flocs [42]. Previous research has found that increasing the number of repeats in these genes linearly increases the adhesion properties of their protein products, as well as the fraction of flocculating cells, which could make cells less accessible to antifungal drugs [43]. The X8 domain (IPR012946; the second top feature) is less well characterized in fungi, but some proteins with this domain are known to be involved in cell wall biosynthesis [44]. The third top feature was the myosin head domain (IPR001609), and some myosins have been demonstrated to regulate membrane permeability of fungi, thereby altering their susceptibility to antifungal drugs [45]. Based on the known biology of these genes, we hypothesize that variation in genomic factors related to the composition and integrity of the cell wall and membrane might impact natural drug resistance by changing both the structure of colonies of different species (flocculin repeats) and altering the accessibility and permeability of the cell membranes to antifungal drugs.

Variation in the presence/absence in InterPro functional annotations is only one of the many dimensions of genomic variation that differentiate *Saccharomycotina* species. For example, amino acid mutations in the Erg11 protein are the most commonly characterized causes of resistance to fluconazole in clinical isolates of most yeast human pathogens [11–14,30,38,46], yet sequence variation is not accounted for in the InterPro dataset. Therefore, we next focused on predicting fluconazole resistance solely from Erg11 amino acid sequence variation across *Saccharomycotina* yeast species.

### Different random forest models yield similar fluconazole resistance prediction accuracies and implicate the same Erg11 sites

**Fig 3:**
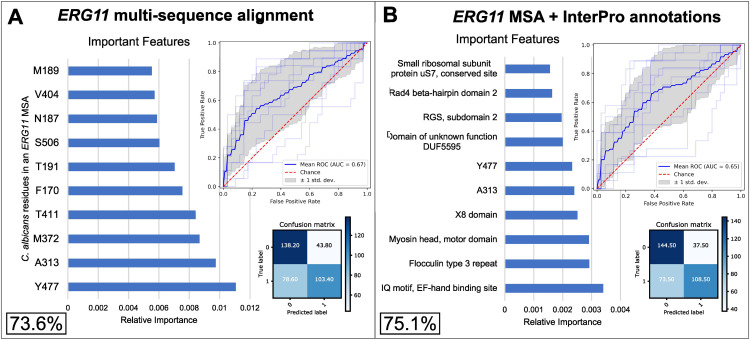
Training a random forest algorithm on the multiple sequence alignment of the known resistance protein Erg11 identifies numerous sites predictive of resistance to fluconazole. Using an integer-encoded multisequence alignment of Erg11 in all *Saccharomycotina* yeasts as input data*,* the random forest algorithm predicted resistance to fluconazole with moderate accuracy **(A)**. Adding in the InterPro annotations slightly increased accuracy, but residues in the alignment remained some of the most important features **(B)**. Accuracy is shown in the form of confusion matrices (matrix in the bottom right in each panel), which show yeasts predicted correctly to be sensitive (true negatives, top left corner of the matrix), yeasts predicted to be resistant but are not (false positives, top right), yeasts correctly predicted to be resistant (true positives, bottom right), and yeasts correctly predicted to be sensitive (false negatives, bottom left). Receiver Operating Characteristic (ROC) curves (top left in each panel) show the true positive rate over false positive rate with changing classification thresholds. Feature importance graphs (left) show the residues or InterPro annotations that are most informative for predicting growth on fluconazole. The accuracies in the bottom left corner of each panel are cross-validated balanced accuracy over 20 down-sampled runs. 527 yeasts had a high-quality hit for an *ERG11* sequence and drug resistance data, but the remaining 5 that had drug resistance data but no high quality *ERG11* sequence were still included in the analysis with an empty input for the *ERG11* sequence.

To test whether variation in specific sites of the Erg11 protein sequence contributed to fluconazole resistance prediction accuracy, we identified and aligned Erg11 orthologs across the yeast subphylum using the MAFFT sequence alignment algorithm [47]. We then trained random forest models to predict fluconazole resistance based on data from (a) both InterPro gene functional annotations and Erg11 MAFFT alignment sites, and (b) just Erg11 MAFFT alignment sites. We found that accuracy of prediction remained similar (75.1% when using both InterPro functional annotations and Erg11 sites, and 73.6% when using just Erg11 sites) (Fig 3). The fact that Erg11 results in similar predictive accuracy as a genome-wide ensemble of functional annotation variation data is consistent with the central role of Erg11 as an azole drug target.

To explore the effects of different methods of aligning and encoding the Erg11 sequence on the training of the random forest algorithm, we used several different methods, including (a) a different sequence alignment algorithm, Muscle5 [48]; (b) one-hot encoding presence and absence of each variant in the alignment; (c) a sequence alignment derived from the superposition of structural models of all Erg11 proteins present in *Saccharomycotina* yeast species; and (d) an alignment-free, k-mer-based (k = 3) approach to encode all Erg11 protein sequences from *Saccharomycotina* yeast species. None of these methods substantially influenced prediction accuracy (S3 Fig). Importantly, all methods identified many of the same sites in the Erg11 protein as the top predictive features: the three different alignment methods (MAFFT, Muscle5, and structural sequence alignment) all identified same top three most informative sites; and, when using one-hot encoding, seven out of top ten variants were located at 5 sites that were also seen in the top 10 sites of all three alignment methods (S3 Fig). Similarly, four of the top five most informative k-mers in the k-mer based method were within two residues of sites in the *C. albicans* Erg11 sequence that were in the top ten most informative sites in the alignment-based methods. These results indicate that diverse methods all identify the same few sites that are most informative for predicting fluconazole resistance.

### Some top sites in Erg11 that predict fluconazole resistance have been experimentally shown to confer resistance

**Fig 4:**
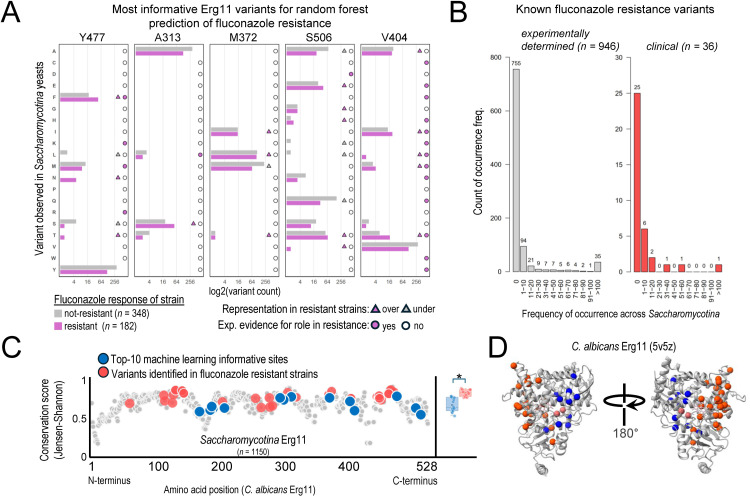
The most informative Erg11 sites have been experimentally shown to confer resistance and were generally less conserved than sites previously found to confer fluconazole resistance in clinical isolates. **(A)** Variant frequencies across Erg11 protein sequences from *Saccharomycotina* yeasts (n = 530) for five of the most informative sites identified by a random forest algorithm trained to predict fluconazole resistance. Over- and under-representation of a given variant is shown when either >44% (over) or <24% (under) of fluconazole-resistant yeasts contained the indicated amino acid substitution. Experimental evidence for fluconazole resistance to each amino acid substitution was taken from Bedard et al. [27]. **(B)** Experimentally verified [27] or clinically characterized amino acid substitution frequencies across *Saccharomycotina*. **(C)** Per-site conservation of all aligned residues of Erg11 across *Saccharomycotina* yeasts. Amino acid positions implicated in fluconazole resistant clinical cases (red) are significantly more conserved than the top ten most informative sites identified by our random forest algorithm (blue) (box plot; p = 0.00024, Mann Whitney U Test). **(D)** Crystal structure of *C. albicans* Erg11 showing spatial distributions of the classifier’s top ten most informative (blue) and clinical resistance-conferring (red) residues. Other views of the crystal structure are shown in S7 Fig.

To examine whether variation at sites predicted by our models can actually confer fluconazole resistance, we examined data from a recent deep mutational scan experiment measuring the effect of individual amino acid substitutions across 206 sites in *C. albicans* Erg11 on fluconazole resistance [27]. Five of our ten most informative sites were tested in these experiments: our top two sites, Y477 and A313, as well as M372, S506, and V404 (numbering based on the *C. albicans* strain CBS 562 protein sequence). Variants at four of those five sites resulted in significantly increased resistance to fluconazole, including variants in the top site, Y477, and in sites A313, S506, and V404 (Fig 4A). While we have not demonstrated that variants in our top sites are causal, the inclusion of several experimentally verified resistance-conferring variants among our informative sites (e.g., Y477F, A313L, and V404T) shows that at least some resistance-conferring variants are being captured by our random forest models.

There are several other natural variants present at these sites that do not appear to confer fluconazole resistance but differ in their frequencies between drug-resistant and -sensitive yeasts; some of these variants are more common in fluconazole-resistant yeasts, while others are more common in fluconazole-sensitive ones. For example, variant A313S was present in the Erg11 sequences of 71 assayed yeasts and coincided with a fluconazole-resistance phenotype 71.8% of the time; conversely, S506Q was present in 191 yeasts and coincided with a fluconazole-susceptibility phenotype 84.2% of the time. Such patterns of variation, where a variant disproportionality associates with either a resistance or susceptibility phenotype, inform our random forest models and are used by them to predict fluconazole resistance (Fig 4A).

### Top Erg11 sites identified by random forest models are more variable and spatially separated from those conferring fluconazole resistance in the clinic

When using the MAFFT Erg11 multiple sequence alignment to predict fluconazole resistance, the ten sites with the highest feature importance corresponded to *C. albicans* Erg11 residues Y477, A313, M372, T411, F170, T191, S506, N187, V404, and M189 (from most important (0.011 relative importance) to least important (0.0055 relative importance)) (Fig 3). Interestingly, none of these residues overlapped with sites harboring 36 Erg11 mutations previously implicated in drug resistance of clinical isolates [11–13,30,37,39–41,46,49–51]. Twenty-five of the 36 sites had zero importance in predicting fluconazole resistance using the MAFFT alignment, and the remaining 11 had relative importances of 0.0038 or less (Fig 4B, S6 and S7 Tables). The minimal contribution of mutations known to confer resistance in clinical settings to our predictions reflects the stronger evolutionary conservation at all these sites (mean Jensen-Shannon divergence (JSD) = 0.76) (S7 Table). In contrast, evolutionary conservation at our top ten most-informative sites was significantly lower (mean JSD = 0.64; p = 0.00024, Mann Whitney U Test; Fig 4C). Only variable sites are expected to be informative for predicting fluconazole resistance in machine learning models, and sites with no variation are simply uninformative for predicting variation in resistance. These results raise the hypothesis that variants contributing to drug resistance in natural isolates across entire lineages may differ substantially from mutations found to confer resistance in specific pathogens in clinical settings.

Mapping the ten most informative residues for predicting fluconazole onto the high-resolution crystal structure of *C. albicans* Erg11 (5v5z) shows that all ten sites cluster separately from previous clinical variants (Figs 4D, S7). This pattern of spatial segregation, considered in conjunction with the higher sequence conservation of sites that harbor clinical variants across Erg11 protein sequences from *Saccharomycotina* yeasts, suggests that structural constraints may be limiting variation at those sites seen almost exclusively in clinical contexts. Indeed, resistance-conferring clinical mutations all occur within 12Å or less of the Erg11 active site or the natively bound heme (both of which are involved in azole binding), and sites in these functionally important regions are less likely to tolerate variation (mean JSD = 0.75 of 213 residues within 12 Å of heme or bound itraconazole in 5v5z). Therefore, differing levels of sequence conservation observed between the ten most informative residues and sites harboring the fluconazole resistance-conferring clinical mutations in *Saccharomycotina* Erg11 proteins may be the result of biophysical constraints in the Erg11 structure itself.

### Erg11 variants informative for predicting fluconazole resistance are less destabilizing than clinical and experimental resistance-conferring variants

**Fig 5:**
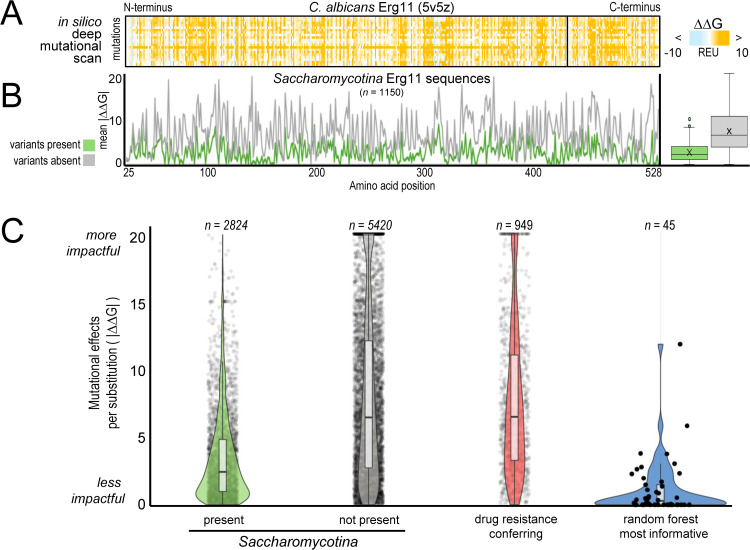
Erg11 variants naturally present in *Saccharomycotina* yeasts, especially those used that best predict fluconazole resistance, are less destabilizing than other Erg11 variants, as well as previously known clinical variants. **(A)** *In silico* deep mutational scan results of the heme-bound form of *C. albicans* Erg11. Heatmap intensities represent the degree to which each amino acid substitution is predicted to affect the stability of the folded protein relative to wild type (DDG). Positive DDG values are destabilizing, and negative DDG values are hyper-stabilizing. **(B)** The mean predicted DDG per site, for just those amino acid substitutions that are either present (green) or wholly absent in 1,150 *Saccharomycotina* yeasts*.* Natural variation is significantly less destabilizing than variation that is never seen (box plot; p = 0.00000000, Mann Whitney U Test). **(C)** Mutational effects for every possible Erg11 mutation. Erg11 variants known to confer fluconazole resistance (red) are significantly more destabilizing than variants naturally present across *Saccharomycotina* (green).

Sites harboring resistance-conferring mutations in the clinic are highly conserved while the ten most informative residues identified by our random forest models are less conserved. This raises the question of whether these two types of sites are evolving under different levels of biophysical constraint. To test this hypothesis, we performed an *in silico* deep mutational scan of *C. albicans* Erg11 to evaluate the impact of every possible amino acid substitution on the predicted structural stability of the Erg11 protein (Fig 5A; Methods). We found that Erg11 amino acid variants observed in natural isolates of *Saccharomycotina* are predicted to have significantly lower mean mutational effects per site (i.e., lower changes in their free energy of folding (i.e., ∆∆G)) compared to variants that are never seen (Fig 5B). This distinction also holds when considering these variants individually, with naturally occurring Erg11 variants being substantially less energetically perturbing than variants that are never observed across *Saccharomycotina* (Fig 5C). These observations are consistent with a model of Erg11 protein sequence evolution where purifying selection acts against variants that substantially disrupt the energetic stability of Erg11.

Interestingly, if we consider known resistance-conferring mutations identified in the clinic or experimentally determined (i.e., fluconazole resistance-conferring variants identified through an experimental deep mutational scan of 206 sites in *C. albicans* Erg11 [27]), they also are significantly more energetically unfavorable than naturally occurring Erg11 variants present in *Saccharomycotina* yeasts; indeed, they are energetically indistinguishable from those variants that are never observed across *Saccharomycotina*. In contrast to the resistance-conferring clinical and experimental mutations, the predicted mutational effects of the 50 most informative variants from the one-hot encoded model are significantly lower and rank among the most energetically conservative variants seen across *Saccharomycotina* yeasts (Fig 5C).

Thus, while biophysical constraints render clinically- or experimentally-determined fluconazole resistance variants uninformative for predicting resistance from natural Erg11 sequences of *Saccharomycotina* yeasts, machine learning approaches can nevertheless leverage natural variation to accurately predict resistance.

### Shared evolutionary history does not explain the association of individual Erg11 variants with fluconazole resistance

One potential explanation for the observed associations is that the top Erg11 sites may simply reflect the phylogenetic relationships of the species tested (even though the pattern of drug resistance itself is not explained by shared evolutionary history; Table 1). To evaluate whether the individual Erg11 amino acid sites identified by the random forest model are not themselves influenced by phylogeny, we applied a logistic model to measure the association of variants at every Erg11 site with the presence/absence of fluconazole resistance, both with and without correcting for phylogenetic relatedness (Methods; S6 Fig).

**Fig 6:**
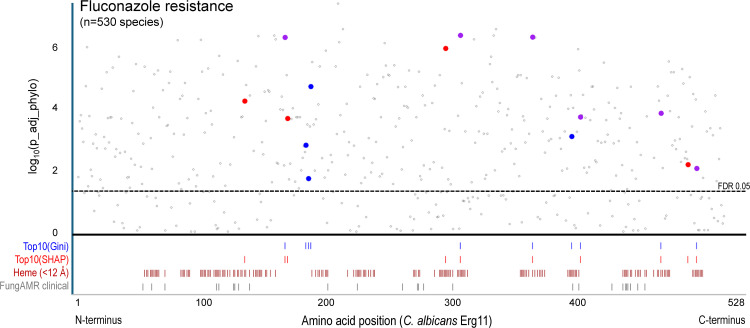
The most informative sites identified by the random forest classifier are robustly associated with fluconazole resistance in a phylogenetic logistic regression. Manhattan plot showing each amino acid position of *C. albicans* Erg11 and the site’s corresponding association with fluconazole resistance following logistic regression with phylogenetic correction. Highlighted are the Top 10 sites from the random forest classifier based upon their Gini- and SHAP-measured feature importance. Also shown are the Erg11 residues that are within 12 Å of the bound heme along with residues specifically implicated in clinical cases of fluconazole-resistant strains of *C. albicans* from the FungAMR database [84].

We next compared these results to the most informative sites identified by our random forest classifier. Among the top 10 informative sites highlighted above (Fig 3A), all sites are significantly associated with fluconazole drug resistance both before and after accounting for phylogeny (Fig 6). We then went on to examine the Erg11 sites ranked among both the top 50 in terms of Gini importance and SHAP values. This set comprised 33 shared sites, only three of which were not statistically significant after accounting for phylogeny (S8 Fig). Moreover, four of the 33 sites are statistically significant only after phylogenetic correction (S7 Fig). Thus, not only do these results confirm that the sites identified by the classifier are robust to phylogenetic non-independence, but they also reveal the surprising finding that the random forest classifier may sometimes even be able to correct for bias in the data resulting from phylogenetic structure (even though phylogenetic information was not explicitly used to train the algorithm).

## Discussion

Examining antifungal resistance across 532 *Saccharomycotina* yeasts has informed our understanding of how resistance may evolve outside of clinical settings. Varying levels of resistance to eight different antifungal drugs were observed throughout the subphylum, and a random forest algorithm was effective in leveraging variation in InterPro functional annotation to predict resistance across hundreds of species of yeasts (each represented by a single strain) with moderate accuracy. Variation in the sizes of gene families that impact cell wall composition and colony structure was the most informative, rather than variation in the sizes of gene families known to be directly involved in drug resistance, which suggests that machine learning can pick up on features that impact resistance, even if their molecular mechanism(s) is indirect.

More than one third of *Saccharomycotina* yeasts tested were resistant to fluconazole. This result was somewhat surprising considering that azoles are synthetic drugs that were developed beginning in the late 1970s [52] and that most of these yeasts were isolated from non-clinical environments. While some of these instances could be due to genomic variation that only incidentally confers azole resistance, azoles have also been widely applied outside of the clinic in agricultural contexts [19]. For example, a recent study found that ~120,000 tons of azoles were sold between 2010 and 2021 just in Europe alone [53]. Consequently, fluconazole is now routinely found in wastewater, groundwater, surface waters, and drinking water worldwide [54], which could foster the evolution of acquired resistance in natural yeast populations.

Fluconazole directly targets Erg11 and point mutations in Erg11 are known to disrupt drug binding. Many previous studies have identified Erg11 mutations in fluconazole-resistant clinical strains of pathogenic yeasts [11–14,30,46]. Therefore, to predict fluconazole resistance, we hypothesized that Erg11 variants in general, and coding variants in particular would be both informative and interpretable. Indeed, an algorithm trained on Erg11 amino acid sequence variation was just as accurate as InterPro functional annotation variation, confirming that compositional variation within the Erg11 protein contributes to prediction of fluconazole resistance. Furthermore, we found that the variants identified by the machine learning classifier were not only confirmed by logistic regression analysis with phylogenetic correction, but that some of these variant sites were only significant after phylogenetic correction was applied. This means that our classifier accounted, at least partially, for the historical relationships between individual Erg11 sequences, possibly by leveraging the co-occurrence of amino acid states.

Importantly, previously identified clinical variants were not informative in our machine learning predictions due to their near complete absence across *Saccharomycotina* yeasts. Indeed, sites containing known, azole resistance-conferring residues were among the least variable sites of Erg11. Rather, the most informative residues to our models were among the more variable sites and appear spatially separated from those known sites in the Erg11 protein structure (Fig 4).

To address why that may be, we turned to an *in silico* deep mutational scanning approach to evaluate the impacts of all possible amino acid substitutions to the structural stability of the Erg11 protein. Biophysical modeling of resistance-conferring variants in Erg11 showed that the energetic costs of natural variants observed across *Saccharomycotina* yeasts were much lower than most of the resistance-conferring variants identified in clinical settings or by *in vitro* deep mutational scanning experiments. These results show how machine learning can leverage natural variation at sites proximal to known resistance-conferring sites to predict resistance across large evolutionary timescales.

Our study raises the hypothesis that the variants associated with natural resistance may be distinct from those that contribute to acquired resistance in the clinic. This idea is supported by the observation that those resistance-conferring, single amino acid Erg11 variants observed exclusively within clinical contexts come at large energetic costs to Erg11 (Figs 4 and 5), and could reflect strong, short-term selective pressures that are rare or absent in natural populations of *Saccharomycotina* yeasts. There is extensive support for this hypothesis in studies of drug resistance in bacteria. For example, natural resistance of bacterial species is typically mediated through genetic changes that are distinct from those that confer acquired resistance [55]. Furthermore, experimental evolution studies of bacteria grown in the presence of an antibiotic have shown how variation in lifestyle selects for resistance mutations in different pathways; whereas experimental evolution of well-mixed bacterial populations results in the selection of resistance mutations in the protein directly targeted by the antibiotic, evolution of biofilm populations results in the selection of resistance mutations that modulate the regulation of efflux pumps [56].

The ecological setting for yeast populations evolving drug resistance in natural versus clinical environments is also likely to differ. Drug resistance in the clinic typically evolves because of an infection by a single isolate that propagates inside a patient. This homogeneous pathogen population will likely be exposed to very high concentrations of the drug for long periods of time and throughout a patient’s body [57], suggesting that evolving resistance to the drug(s) used to treat the infection is likely to be a main, if not the main, selective agent. In such an environment, a mutation that confers resistance but destabilizes the protein targeted by the drug could be strongly favored. For example, multiple studies in *C. albicans* point to azole-resistance conferring variants being moderately-to-severely compromised in their normal catalytic activity [37,40,58,59]. In contrast, an environmental yeast is likely to be simultaneously exposed to many more drugs (produced by other microbes), each of which is at a much lower concentration [60], as well as to other biotic or abiotic factors. In such a complex environment, large-effect mutations that destabilize protein function would likely be selected against. Rather, natural resistance is more likely to involve mutations of small effect that optimize trade-offs between resistance and protein function.

Our study showed that the prediction accuracy values were higher for some drugs than for others, although it is fair to say that none of our models is highly accurate. Drug resistance is a complex trait, so it is possible that decision-based trees trained on genomic features alone may require much more sampling and validation to fully predict drug resistance traits. While we sampled broadly across *Saccharomycotina*, we did not account for within species variability (i.e., strain heterogeneity) in drug resistance. We also only accounted for a small fraction of the genomic features present in yeast genomes (e.g., we did not examine variation in non-coding regions), nor did we account for the limitations and biases associated with InterPro functional annotations. Notwithstanding these caveats, we find it encouraging that by examining only variation in the number of protein domains (and amino acid variants in the case of Erg11) in a relatively small sample we were able to achieve the accuracies we did.

Future studies could sample both within- and between-species genomic variation (not just of protein domains but of other genomic features, such as non-coding regions and codon usage bias) for larger numbers of strains and species from both clinical and environmental settings. Additional phenotypic data (e.g., cell wall thickness, ability to form biofilms) would also be highly informative. Importantly, our machine learning and phylogenetic association tests simply require genome sequence data and drug resistance data for many different organisms; whether these organisms are strains within a species or come from different species does not affect our models or our findings. Of course, inclusion of *intraspecific* variation would provide additional power to our approach (and so would inclusion of data from additional species), since the more data the better. Additionally, the clinical relevance of our classifier could be tested on samples exclusively from clinical settings.

Ultimately it would be desirable to link back the signals identified by our classifier to function, and to interrogate the extent to which informative residues, genes, or functional domains can be related directly back to resistance. As we note above, this would be a non-trivial task due to the likely very complex nature of this trait. But even focusing on informative variants identified in the drug target itself would be difficult because of the likely small effect size that any single site might have and the differing genetic backgrounds of individual species. Even in well-established experimental systems, differences between the genomic background of the model species and that of the gene of interest are known confounders [61–66]. This challenge will only become more pronounced considering the broad evolutionary distances present among *Saccharomycotina*.

In 2009, multidrug-resistant isolates of a novel pathogen, *C. auris*, were near-simultaneously identified in multiple continents [67]; *C. auris* has continued its global spread since and is now considered a critical priority fungal pathogen by the World Health Organization [68]. Although *C. auris* has been detected in various environments, these environmental strains are genomically very similar to clinical strains, suggesting that the organism’s true environmental reservoir(s) remains elusive [69]. The case of *C. auris* emphasizes the importance of understanding the ecology and evolution of lineages harboring fungal pathogens [70]. If the arguments raised here hold, it follows that the evolutionary pathways to drug resistance are likely to differ between clinical and natural isolates and that studies of resistance in both *natural* and *clinical* settings are important. For example, large scale analyses of entire lineages can capture natural variation and highlight evolutionary pathways to drug resistance that may be challenging to discover through studies of acquired resistance in the clinic. We argue that a full understanding of antifungal drug resistance will require examination of both acquired resistance in clinical isolates of yeast pathogens and natural resistance in populations of diverse yeast species that are never encountered in the clinic.

## Methods

### Strain selection

The goal of our study was to characterize variation in resistance across the diversity of yeast species in the *Saccharomycotina* subphylum. Given the large number of yeast species, only one strain was typically examined per species (usually the type strain). Note that we have genomic, metabolic, and isolation environment data from 1,154 yeast strains (representing at least 1,051 species) [1]. From those 1,154 yeasts, we were able to retrieve drug resistance data for 532 through the study of Desnos-Ollivier et al. [9] (see also Antifungal resistance data matrix section below).

### Genomic data matrix

Using InterProScan gene functional annotations generated by the Y1000 + Project [1], a data matrix was built with counts of each unique InterPro ID number in each genome (https://figshare.com/s/739a5de80d5ce89dbd10). Each genome was its own row, and the number of each InterPro ID (*N* = 12,242) present in one or more of the 1,154 yeast genomes was its own column. A python script recorded the number of each InterPro ID for each genome and put them in the appropriate cells of the data matrix.

### Metabolic data matrix

Our metabolic data matrix contained 122 traits from 893 yeast strains (out of the 1,154 total) from 885 species in the subphylum [1,71] (S4 Table). The list of traits in the data matrix included growth on different carbon and nitrogen sources, such as galactose, raffinose, and urea, as well as on environmental conditions, such as growth at different temperatures and salt concentrations. The percentage of missing data in the data matrix was 37.5% (40,906 missing values out of 108,946 total). Less thoroughly studied traits tended to have more missing data than more commonly found and/or thoroughly studied traits.

### Environmental data matrix

The isolation environments for 1,088 (94%) out of the 1,154 yeasts examined were gathered from strain databases, species descriptions, or from *The Yeasts: A Taxonomic Study* [1,72] (S5 Table) and converted into a hierarchical binary trait matrix using a controlled vocabulary containing all the unique environmental descriptors [31]. Strains without isolation environments were either domesticated via crossing or subculturing or lacked information in our searches. The ontology contains six broad isolation environment categories: animal, plant, environmental, fungal, industrial products, and victuals (food or drink). Within these categories, more specific controlled vocabulary annotations are connected to each strain: for example, an isolation environment reported as “*Drosophila hibisci* on *Hibiscus heterophyllus*” is associated in our ontology with the animal subclass “*Drosophila hibisci*” and the plant subclass “*Hibiscus heterophyllus*”.

### Gene sequence data matrix

To retrieve the Erg11 protein sequence(s) from each genome, we used HMMR3 (version 3.1b2) hmmsearch [73]. The sequence alignment profile was constructed with hmmbuild [73] from several documented copies of Erg11 in different species across *Saccharomycotina* yeasts. Four of the 1,154 yeasts had annotated copies of Erg11 that were highly divergent and aligned poorly with the others. The sequences of these four yeasts were therefore excluded from our subsequent analyses. MAFFT version 7 [47] was then used to align the amino acid sequences of Erg11 from the remaining 1,150 yeasts across the subphylum. The resulting multiple sequence alignment was integer-encoded, with each amino acid as well as gaps in the alignment being represented by a different integer. The alignment was then converted into a data matrix where each column represented a different site of the alignment, and each column represented each species. In cases where two copies were found in a genome, the one with the highest sequence similarity score to the HMM profile used to search for Erg11 was used. For the four yeasts with the highly divergent Erg11 sequences, their rows in the dataset were left empty. To examine the impact of different multiple sequence alignment methods on the accuracy and the identification of the most important sites of our classifier, we also generated Erg11 sequence alignments using Muscle5 [48] and protein structure-guided approach (see section on Erg11 structural alignment below).

### Antifungal resistance data matrix

Our drug resistance data matrix contained eight traits from 532 yeasts in the subphylum. The data were sourced from information available for each of the sequenced strains from the CBS strain database. These data were gathered from strains studied as part of the published descriptions of species, additional data on strains obtained by previous studies done in the Westerdijk Fungal Biodiversity Institute (CBS), or additional data provided by the depositors of the strains in the CBS culture collection.

The methods for determining whether a strain was resistant are described in Desnos-Oliver et al. [9]; briefly, drug resistance was assessed for each strain using a microdilution technique according to the procedure and criteria established by the Antifungal Susceptibility Testing Subcommittee of EUCAST (AFST-EUCAST) [9]. MIC was measured as a 50% (or 90% for amphotericin B) reduction in growth compared to the strain grown in a drug-free well. As suggested by EUCAST recommendations, for fluconazole an MIC higher than or equal to 8 µg/mL was defined as resistance, while for voriconazole it was an MIC higher than 0.25 µg/mL. Previous research looking at mutations in target genes was used to determine that an MIC of over 8 µg/mL would be considered resistant for caspofungin and 0.5 µg/mL. Finally, for the remainder of the drugs, the resistance threshold was determined by the MIC at which 90% of clinical isolates were inhibited: ≥ 0.5 µg/mL for itraconazole and posaconazole, ≥ 0.25 µg/mL for Amphotericin B, and ≥8 µg/mL for Terbinafine.

### Classifying resistance to different antifungals using machine learning algorithms trained on genomic, metabolic, and/or environmental data

To test whether we could classify resistance to eight different antifungal drugs from genomic, metabolic, and isolation environment data, we used a random forest algorithm. For each resistance profile, we trained the algorithm separately on a given dataset to evaluate the accuracy of classification and identify the most important predictive features. Although the task being performed is classification, and the random forest algorithm that we use is a classifier, we refer to the results of these analyses throughout this study as “predictions” for ease of understanding.

We trained a machine learning algorithm built by an XGBoost (1.7.3) [74] random forest classifier (XGBRFClassifier()) with the parameters max_depth = 12 and n_estimators = 100; all other parameters were in their default settings. The max_depth parameter specifies the depth of each decision tree, determining how complex the random forest will be to prevent overfitting while maintaining accuracy. The n_estimators parameter specifies the number of decision trees in the forest. After testing the increase in accuracy while increasing each of these parameters, we found that having a higher max_depth or more decision trees per random forest did not further increase accuracy.

Since drug resistance is typically relatively rare, our datasets tended to be highly unbalanced. Before training the random forest algorithm, down-sampling by randomly choosing an equal number of non-resistant species as resistant species was first employed to balance the datasets. The random forest algorithm was then trained on 90% of the data, and used the remaining 10% for cross-validation, using the RepeatedStratifiedKFold and cross_val_score functions from the sklearn.model_selection (1.2.1) package. Cross validation is a method for assessing accuracy involving 10 trials, each of which holds back a random 10% of the training data for testing. We also used the cross_val_predict() function from Sci-Kit Learn separately to generate the confusion matrices; these matrices show the numbers of strains correctly predicted to be resistant or sensitive to a specific antifungal drug (true positives and true negatives, respectively) and incorrectly predicted (false positives, predicted to be resistant but are in reality sensitive; and false negatives, predicted to be sensitive but are in reality resistant). This function also employs a 10-fold cross validation step, but it keeps track of which species are classified as true/false positives and true/false negatives during each of these 10 trials, while entering the final results into a confusion matrix. Top features were automatically generated by the XGBRFClassifier function using Gini importance, which uses node impurity (the amount of variance in resistance for strains that either are or are not resistant to this drug). All these metrics, as well as total balanced accuracy (for which 50% would be equivalent to randomly guessing), were recorded and saved, and then the process was repeated 20 times with new randomly chosen down-sampled datasets each time to account for variation in the yeasts chosen to represent examples of drug-sensitive strains.

Receiver Operating Characteristic (ROC) curves, which plot the true positive rate against the false positive rate, were also generated for each prediction analysis to visualize the accuracy of the algorithm in predicting resistance to a given drug; values of the area under the curve (AUC) greater than 0.5 in these plots indicate better than random classification. Non-down-sampled datasets were used for this analysis, to fully capture the error in the whole dataset. As above, a 10-fold cross validation step was employed such that the model was tested and trained on different subsets on the dataset for each run, i.e., the model was never tested on the data used for its training.

### Erg11 sequence conservation

The Jenson-Shannon entropy metric of protein sequence conservation was generated from the MAFFT MSA using score_conservation.py [75].

### Structural alignments of Saccharomycotina Erg11

Hypothetical structural models for all Erg11 proteins found in the Y1000 + Project genomic dataset from the *Saccharomycotina* subphylum were generated using ESMFold as implemented by ColabFold (v.1.5). ESMFold was chosen over other alternative methods (e.g., over methods such as AlphaFold or homology modeling) for its greater speed and comparable accuracy [76]. A structural MSA was generated from the resulting ESMfold protein models using FoldMason (foldmason easy-msa --report-mode 1 --refine-iters 5) [77].

### *C. albicans* Erg11 protein structure

A protein structure for the apo form of *C. albicans* Erg11 was retrieved from the PDB (5v5z) [78]. The amino acid sequence of the structure was checked and edited to match the Erg11 sequence of the *C. albicans* strain CBS 632 present in the Y1000 + Project (only one amino acid difference was amended). All protein model images were generated using ChimeraX (v1.7) [79].

### *C. albicans* Erg11 *in silico* deep mutational scanning

The apo form of *C. albicans* Erg11 (5v5z) was relaxed in complex with the native heme using Rosetta 3.13. Briefly the structure was cleaned and renumbered using clean_pdb.py and pdb_renumber.py. The cleaned structure was minimized with heme (-nstruct 20, -relax:cartesian true, -default_max_cycles 200), and the lowest energy structure was chosen.

All-way, *in silico* mutagenesis (deep mutational scanning) was conducted using Rosetta 3.13 (cartesian_ddg) and energy minimization protocols and parameterizations previously benchmarked to optimize replication of experimental ΔΔG (DDG) measurements (parser:protocol cartesianrelaxprep.xml) [80]. Three replicates were performed for each substitution (ddg::iterations 3) and the mean change of free energy of folding (ΔG) was derived from the mean difference between wild type and each amino acid substitution (ΔΔG = ΔG (mutant) - ΔG (wild type)) across replicates.

### Phylogenetic signal analysis

The strength of the phylogenetic signal for drug resistance was evaluated using the D measure developed by Fritz and Purvis [35], as implemented in the phylo.d function that is part of the caper (v 1.0.3) R package [81]. The strength of the phylogenetic signal was measured separately for each antifungal drug on a pruned species tree. The D measure statistically evaluates whether the distribution of a trait on a phylogeny is best explained from shared, phylogenetic history (“Brownian”) or from random occurrences over the tree (“random”).

### Association testing

To test for associations between amino acid state and trait state, we began with the Erg11 multiple sequence alignment generated using the MAFFT alignment. Gaps and ambiguous characters in the alignment (e.g., “-”, “X”) were treated as missing data and excluded from the association testing performed at each site. Then, for each aligned position in the Erg11 alignment, the observed amino-acid states were treated as categorical predictors (site_state) with a baseline set to the most frequent amino acid observed at that site, while the trait state was a binary vector representing all the species (trait). We then fit both a standard logistic regression (glm(trait ~ site_state)) and a phylogenetic logistic regression (phyloglm(trait ~ site_state, species_tree, MPLE)) to these data [82]. For each model, effect and significance per site were assessed by a likelihood ratio test (LRT), resulting in a p-value for each of the two tests (p_std for glm; p_phylo for phyloglm). All p-values were subsequently corrected for multiple testing using Benjamini–Hochberg (BH) applied separately to p_std and p_phylo, producing p_adj_std and p_adj_phylo. The p_adj_std and p_adj_phylo values were then used to characterize each site for phylogenetic independence as follows:

“Robust association”: p_adj_std < 0.05 and p_adj_phylo < 0.05
“Explained by phylogeny”: p_adj_std < 0.05 and p_adj_phylo ≥ 0.05
“Revealed by phylogeny”: p_adj_std ≥ 0.05 and p_adj_phylo < 0.05.

## Supporting information

S1 Fig A random forest algorithm weakly predicts fluconazole resistance from environmental and metabolic traits. Accuracy is shown in the form of confusion matrices (bottom right of each panel), which show yeasts predicted correctly to be sensitive to fluconazole (true negatives, top left corner of the matrix), yeasts predicted to be resistant but are not (false positives, top right), yeasts correctly predicted to be resistant (true positives, bottom right), and yeasts correctly predicted to be sensitive (false negatives, bottom left). Receiver Operating Characteristic (ROC) curves (top right of each panel)) show the true positive rate over false positive rate with changing classification thresholds. Feature importance graphs (left of each panel) show the environmental and metabolic features that are most useful for predicting growth on fluconazole. The accuracy in the bottom left corner of each graphic is cross-validated balanced accuracy over 20 down-sampled runs. The environmental features are from an ecological ontology used to describe the isolation environment of each yeast strain [31] and therefore the features are described in relation to each other. For example, “has value” was added as a feature when an additional qualitative descriptor was present in the description of the isolation environment; for example, a strain could be described as found in an environment that “Has_Value”: “HAS_Cooked_food_processing”. The presence of “Has_Value” in the list of most important features suggests the presence of additional descriptors of the isolation environment is useful for predicting drug resistance.
(TIF)

S2 Fig A random forest algorithm predicts resistance to eight antifungal drugs with moderate accuracy from variation in InterPro functional annotations. Accuracy is shown in the form of confusion matrices on the bottom right of each panel, which show yeasts predicted correctly to be sensitive (true negatives, top left of each matrix), yeasts predicted to be resistant but are not (false positives, top right), yeasts correctly predicted to be resistant (true positives, bottom right), and yeasts correctly predicted to be sensitive (false negatives, bottom left). Receiver Operating Characteristic (ROC) curves (top right of each panel) show the true positive rate over false positive rate with changing classification thresholds. The bottom left of each panel corresponds to the average cross-validated balanced accuracy over 20 down-sampled runs. Feature importance graphs (left of each panel) show the InterPro annotations that are most useful for predicting growth on the two drugs. Note that the most informative genomic features were not linked to known drug resistance genes.
(TIF)

S3 Fig Different Erg11 alignments and ways of encoding sequence information are similarly accurate for predicting fluconazole resistance and highlight the same sites. Accuracy is shown in the form of confusion matrices (bottom right of each panel), which show yeasts predicted correctly to be sensitive to fluconazole (true negatives, top left of each matrix), yeasts predicted to be resistant but are not (false positives, top right), yeasts correctly predicted to be resistant (true positives, bottom right), and yeasts correctly predicted to be sensitive (false negatives, bottom left). Receiver Operating Characteristic (ROC) curves (top right of each panel) show the true positive rate over false positive rate with changing classification thresholds. The accuracy in the bottom left corner of each graphic is cross-validated balanced accuracy over 20 down-sampled runs. Feature importance graphs (left of each panel) show the sites and variants are most useful for predicting resistance to fluconazole. In panel B, the dash symbol (“-“) indicates that the informative *C. albicans* Erg11 position was represented by a gap in the Erg11 multiple sequence alignment.
(TIF)

S4 Fig Resistance profiles to antifungal drugs vary throughout the *Saccharomycotina* subphylum. Dark colors denote resistance, light colors denote susceptibility, and no color denotes absence of testing. Yeast names are included. The colors of the different branches of the phylogeny correspond to the 12 taxonomic orders [1,83]. Drug resistance data obtained using the microdilution technique described in Desnos-Ollivier et al. 2012 [9]. Note that this visualization of antifungal drug resistance profiles does not consider within-species variation in drug resistance.
(TIF)

S5 Fig SHAP values identify many of the same features as Gini impurity when used to assess feature importance in a random forest mode predicting fluconazole resistance using an *ERG11* alignment or InterPro annotations. 10-fold cross-validated models that were not down-sampled were used for ease of comparison.
(TIF)

S6 Fig Erg11 sites associated with fluconazole resistance across *Saccharomycotina* yeasts do not simply reflect phylogeny. Manhattan plot showing each amino acid position of *C. albican*s Erg11 and the corresponding associations of amino acid variation (n = 530 phenotyped strains) with fluconazole resistance both without (top) and with (bottom) phylogenetic correction using the *Saccharomycotina* species phylogeny. In both cases, p-values have been corrected for multiple testing (Benjamini Hochberg).
(TIF)

S7 Fig Distribution of clinical and random forest informative variants on different views of the Erg11 structure of *C. albicans.* Information being presented is identical to that shown in Fig 4D.
(TIF)

S8 Fig Counts of Erg11 sites significantly associated with fluconazole resistance. Shown are the counts of those variants that appear among those highly informative to the random forest classifier and those significant under a logistic model with phylogenetic correction. Note that “Revealed by phylogeny” and “Robust to phylogeny” categories are by their definition, mutually exclusive (Methods).
(TIF)

S1 Table Isolation environments of each mammalian-associated yeast in the antifungal drug resistance dataset. (XLSX)

S2 Table Resistance to eight different antifungal drugs for 532 species of *Saccharomycotina* yeasts. (XLSX)

S3 Table Frequency of resistance to each antifungal drug in each order of *Saccharomycotina.* (XLSX)

S4 Table Accuracy of predicting resistance to each antifungal drug when a random forest algorithm is trained on metabolic or environmental datasets. (XLSX)

S5 Table Average most informative features when a random forest algorithm is trained on metabolic or environmental datasets to predict resistance to eight different antifungal drugs. (XLSX)

S6 Table Gini importance of each site in the MAFFT alignment of all Erg11 protein sequences across *Saccharomycotina* yeasts, which *C. albicans* residue they correspond to (if any), and whether (*) that site has been previously observed in clinical isolates. (XLSX)

S7 Table All 36 clinical variants known to conference resistance, the study that identified them, their count numbers in the Y1000+ Project dataset, and their Gini importance for each different method of encoding Erg11. (XLSX)
