# Supplementary figures and images for: Machine learning identifies novel signatures of antifungal drug resistance in Saccharomycotina yeasts
Source: PLoS Genet. 2026 Mar 17;22(3):e1012091. doi: 10.1371/journal.pgen.1012091 (PMC13012505; doi:10.1371/journal.pgen.1012091)

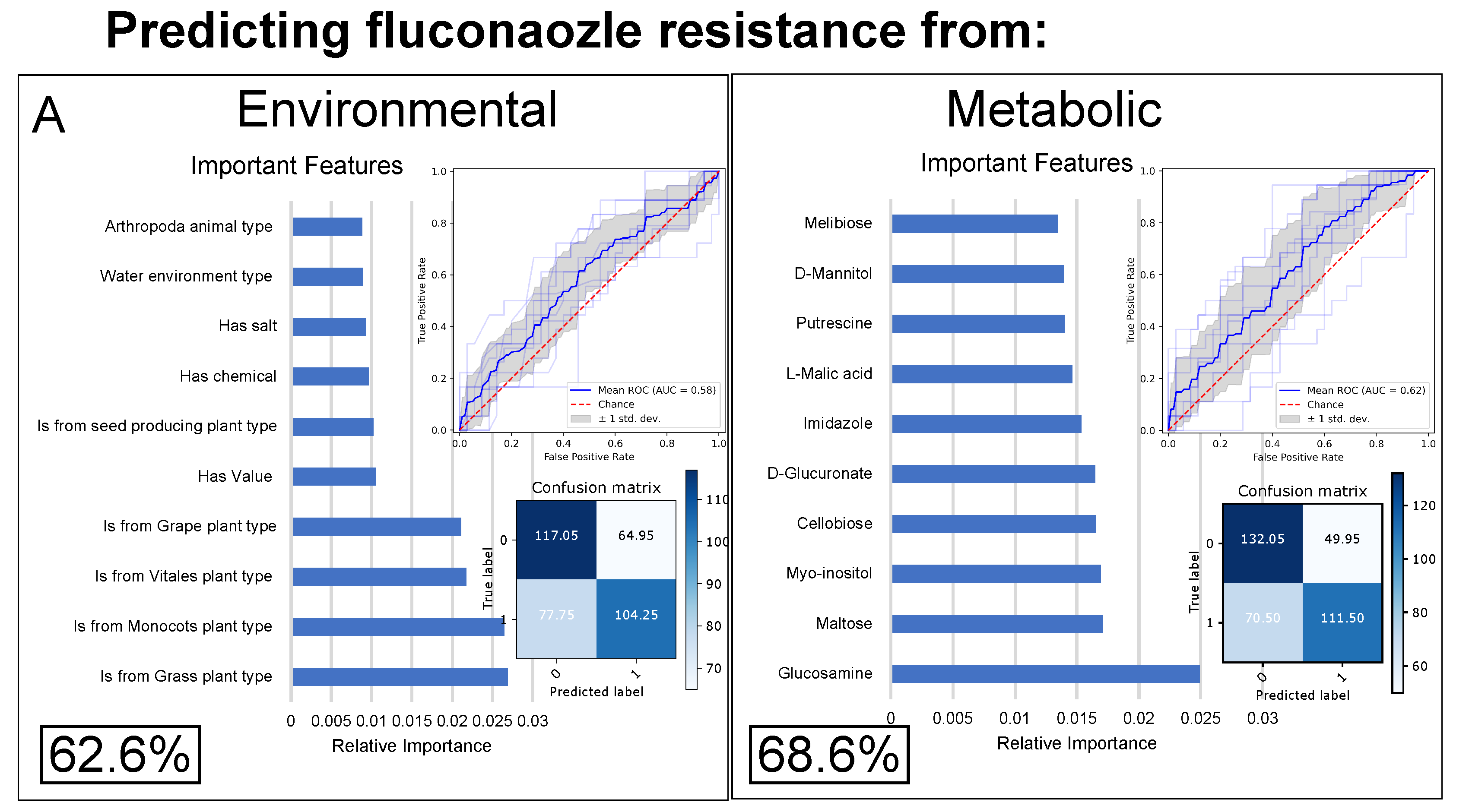

Supplement: S1 Fig — Accuracy is shown in the form of confusion matrices (bottom right of each panel), which show yeasts predicted correctly to be sensitive to fluconazole (true negatives, top left corner of the matrix), yeasts predicted to be resistant but are not (false positives, top right), yeasts correctly predicted to be resistant (true positives, bottom right), and yeasts correctly predicted to be sensitive (false negatives, bottom left). Receiver Operating Characteristic (ROC) curves (top right of each panel)) show the true positive rate over false positive rate with changing classification thresholds. Feature importance graphs (left of each panel) show the environmental and metabolic features that are most useful for predicting growth on fluconazole. The accuracy in the bottom left corner of each graphic is cross-validated balanced accuracy over 20 down-sampled runs. The environmental features are from an ecological ontology used to describe the isolation environment of each yeast strain [31] and therefore the features are described in relation to each other. For example, “has value” was added as a feature when an additional qualitative descriptor was present in the description of the isolation environment; for example, a strain could be described as found in an environment that “Has_Value”: “HAS_Cooked_food_processing”. The presence of “Has_Value” in the list of most important features suggests the presence of additional descriptors of the isolation environment is useful for predicting drug resistance. (TIF) [file pgen.1012091.s001.tif]

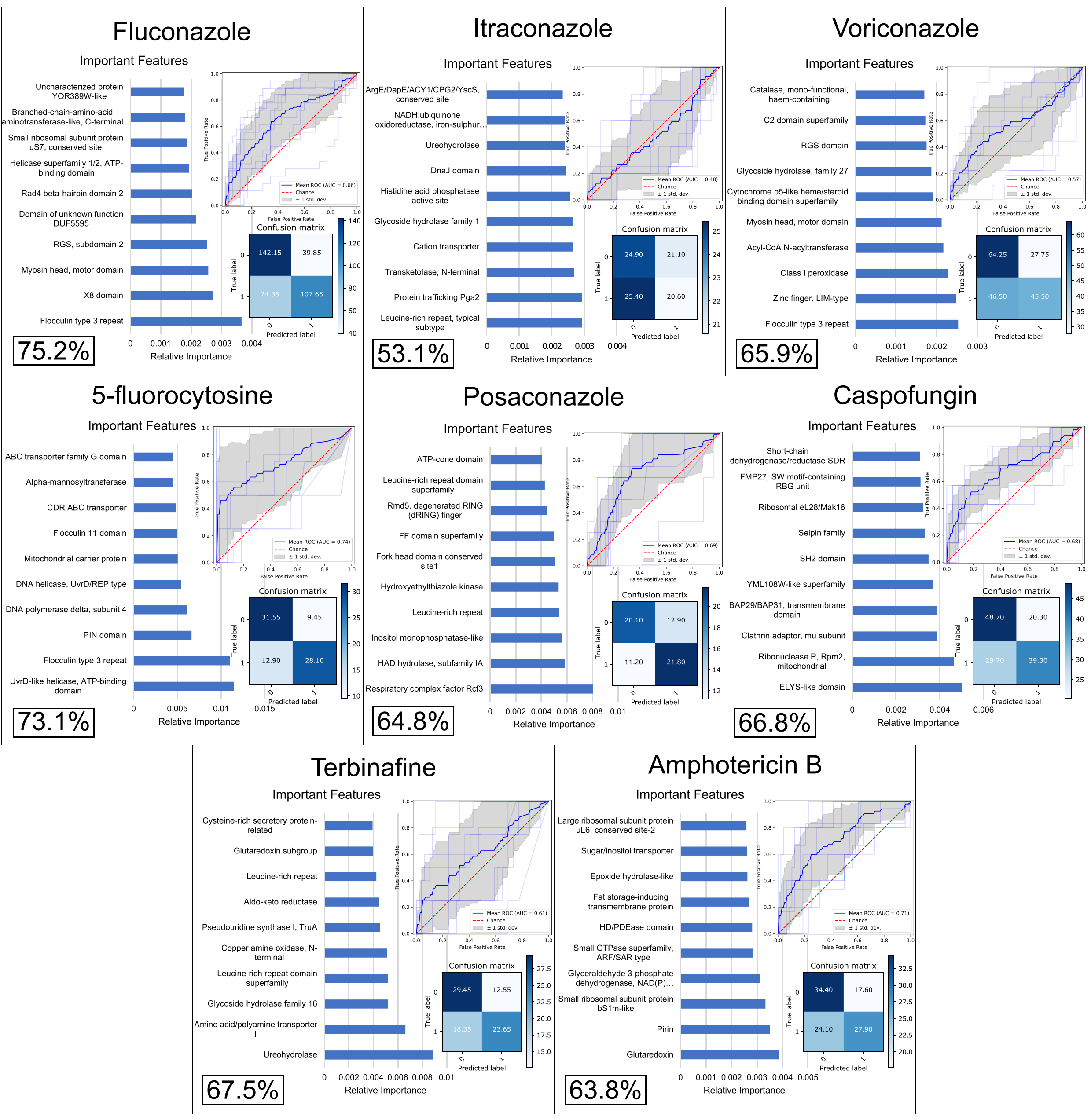

Supplement: S2 Fig — Accuracy is shown in the form of confusion matrices on the bottom right of each panel, which show yeasts predicted correctly to be sensitive (true negatives, top left of each matrix), yeasts predicted to be resistant but are not (false positives, top right), yeasts correctly predicted to be resistant (true positives, bottom right), and yeasts correctly predicted to be sensitive (false negatives, bottom left). Receiver Operating Characteristic (ROC) curves (top right of each panel) show the true positive rate over false positive rate with changing classification thresholds. The bottom left of each panel corresponds to the average cross-validated balanced accuracy over 20 down-sampled runs. Feature importance graphs (left of each panel) show the InterPro annotations that are most useful for predicting growth on the two drugs. Note that the most informative genomic features were not linked to known drug resistance genes. (TIF) [file pgen.1012091.s002.tif]

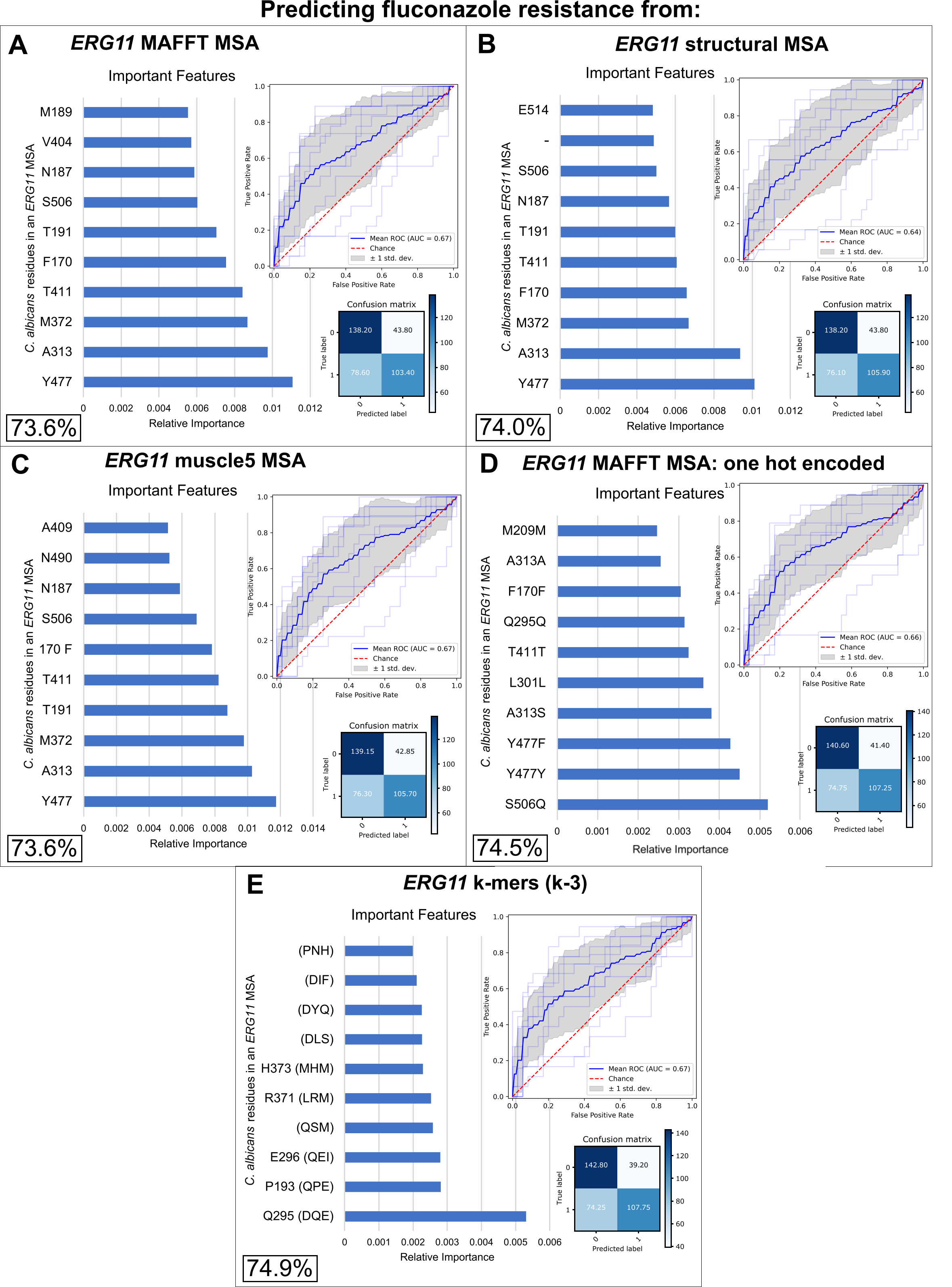

Supplement: S3 Fig — Accuracy is shown in the form of confusion matrices (bottom right of each panel), which show yeasts predicted correctly to be sensitive to fluconazole (true negatives, top left of each matrix), yeasts predicted to be resistant but are not (false positives, top right), yeasts correctly predicted to be resistant (true positives, bottom right), and yeasts correctly predicted to be sensitive (false negatives, bottom left). Receiver Operating Characteristic (ROC) curves (top right of each panel) show the true positive rate over false positive rate with changing classification thresholds. The accuracy in the bottom left corner of each graphic is cross-validated balanced accuracy over 20 down-sampled runs. Feature importance graphs (left of each panel) show the sites and variants are most useful for predicting resistance to fluconazole. In panel B, the dash symbol (“-“) indicates that the informative C. albicans Erg11 position was represented by a gap in the Erg11 multiple sequence alignment. (TIF) [file pgen.1012091.s003.tif]

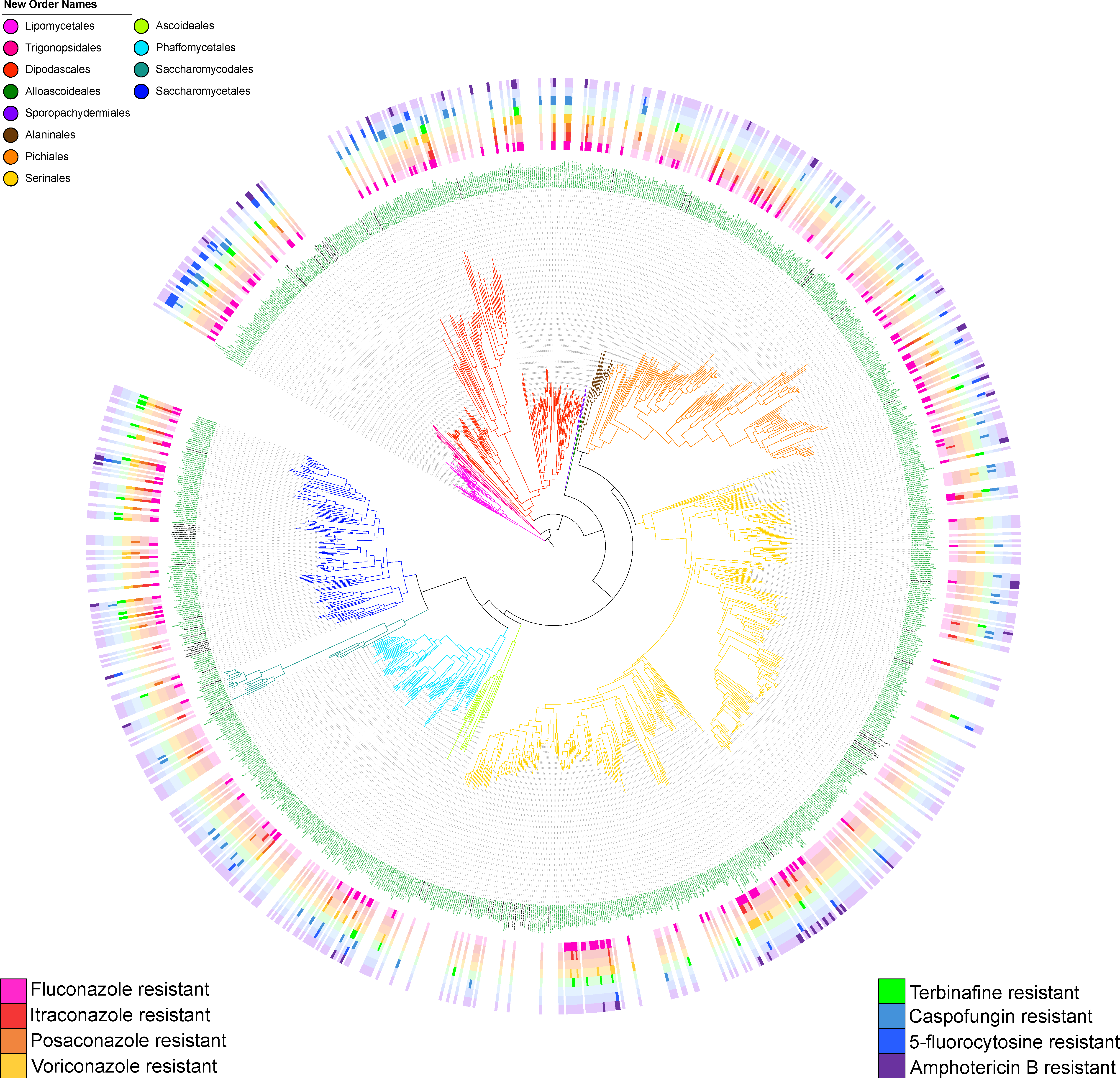

Supplement: S4 Fig — Dark colors denote resistance, light colors denote susceptibility, and no color denotes absence of testing. Yeast names are included. The colors of the different branches of the phylogeny correspond to the 12 taxonomic orders [1,83]. Drug resistance data obtained using the microdilution technique described in Desnos-Ollivier et al. 2012 [9]. Note that this visualization of antifungal drug resistance profiles does not consider within-species variation in drug resistance. (TIF) [file pgen.1012091.s004.tif]

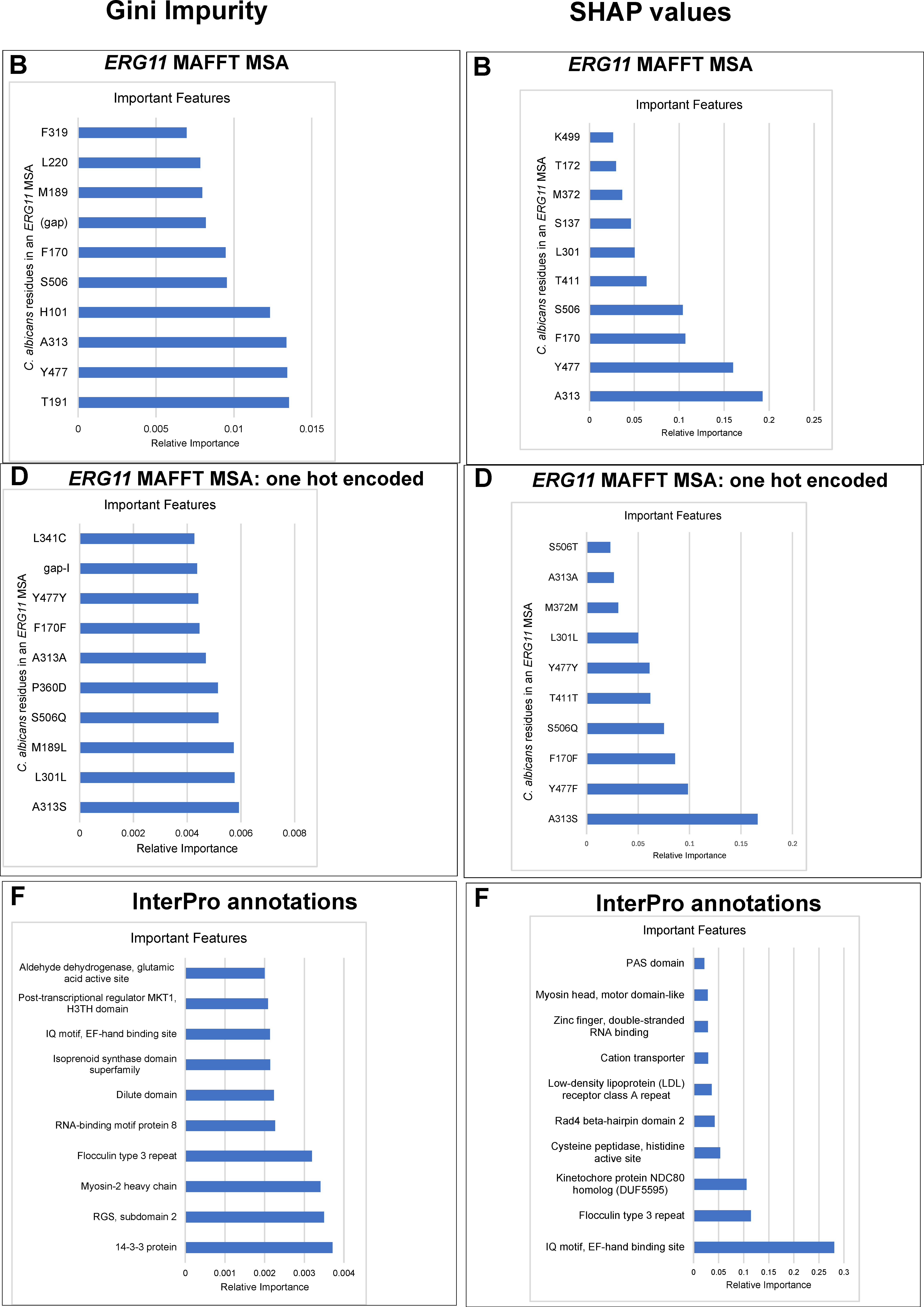

Supplement: S5 Fig — 10-fold cross-validated models that were not down-sampled were used for ease of comparison. (TIF) [file pgen.1012091.s005.tif]

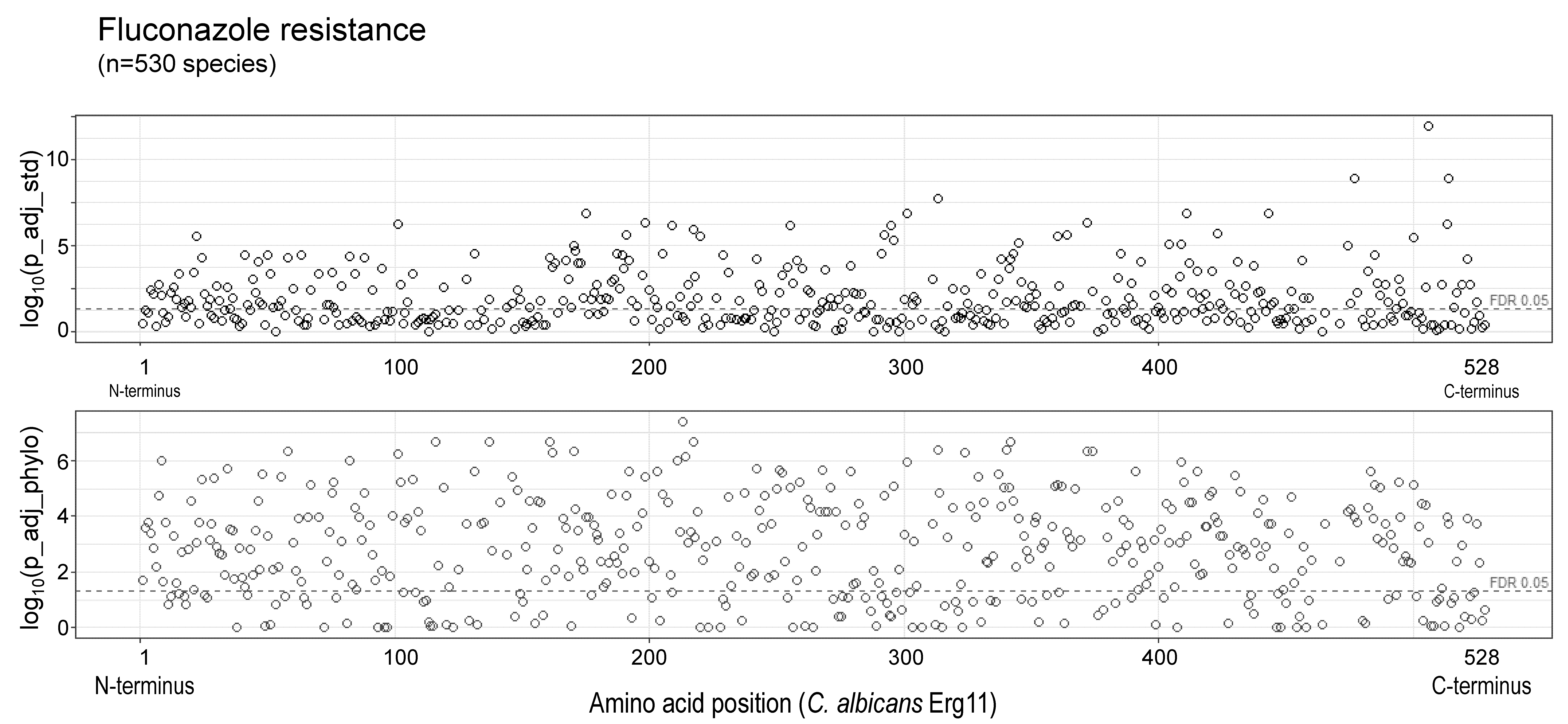

Supplement: S6 Fig — Manhattan plot showing each amino acid position of C. albicans Erg11 and the corresponding associations of amino acid variation (n = 530 phenotyped strains) with fluconazole resistance both without (top) and with (bottom) phylogenetic correction using the Saccharomycotina species phylogeny. In both cases, p-values have been corrected for multiple testing (Benjamini Hochberg). (TIF) [file pgen.1012091.s006.tif]

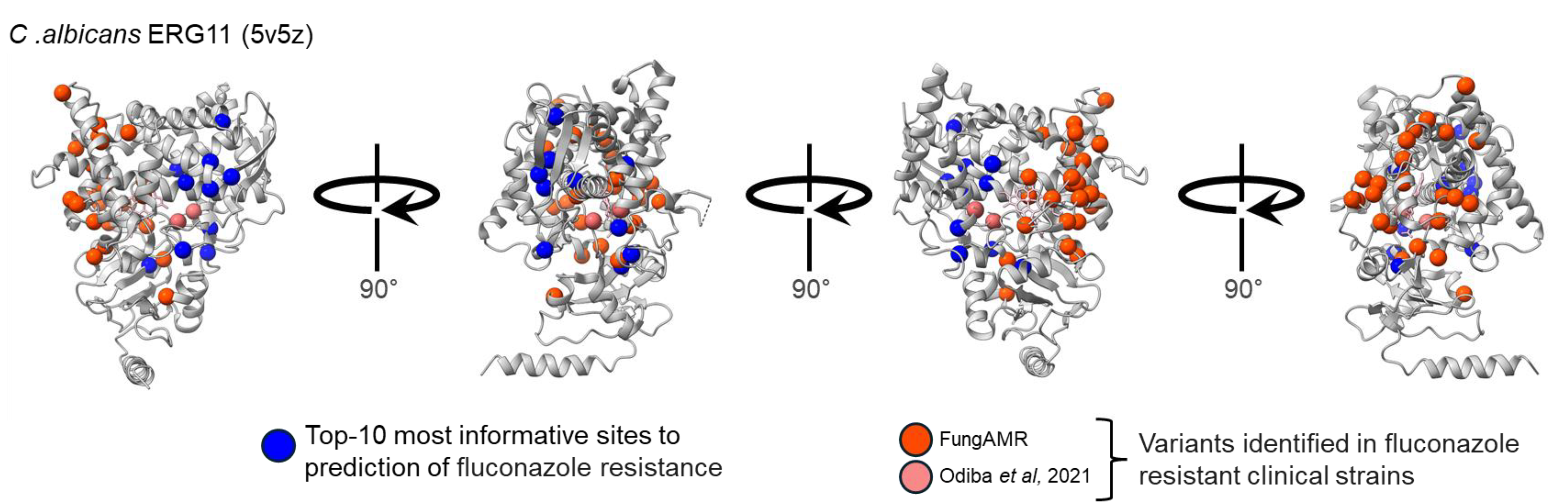

Supplement: S7 Fig — Information being presented is identical to that shown in Fig 4D. (TIF) [file pgen.1012091.s007.tif]

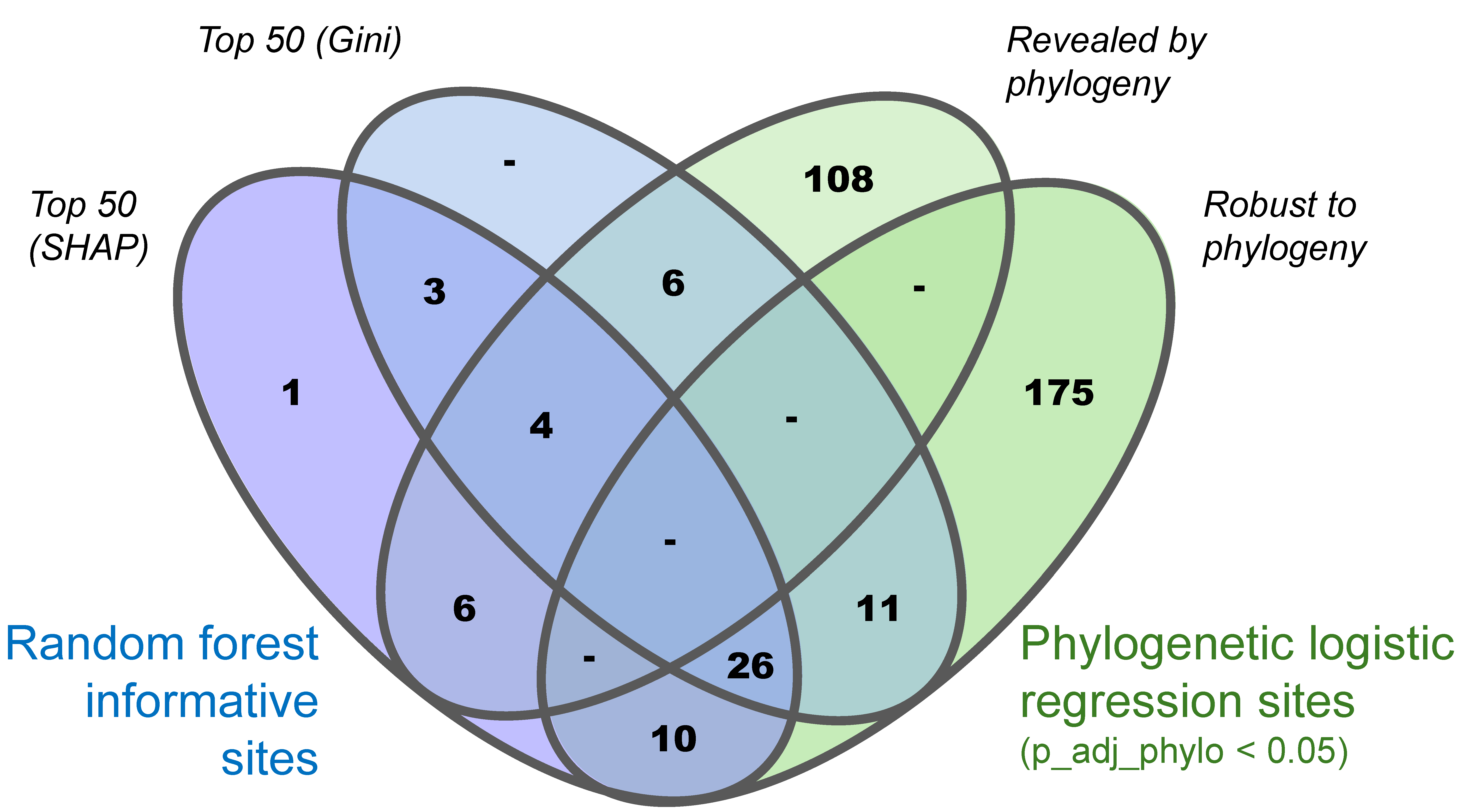

Supplement: S8 Fig — Shown are the counts of those variants that appear among those highly informative to the random forest classifier and those significant under a logistic model with phylogenetic correction. Note that “Revealed by phylogeny” and “Robust to phylogeny” categories are by their definition, mutually exclusive (Methods). (TIF) [file pgen.1012091.s008.tif]
